# Increased risk of hearing loss associated with macrolide use: a systematic review and meta-analysis

**DOI:** 10.1038/s41598-023-50774-1

**Published:** 2024-01-02

**Authors:** Sung Ryul Shim, YungJin Lee, Seung Min In, Ki‐Il Lee, Ikhee Kim, Hyoyeon Jeong, Jieun Shin, Jong-Yeup Kim

**Affiliations:** 1 Department of Biomedical Informatics, College of Medicine, Konyang University, Daejeon, Republic of Korea; 2 Konyang Medical data Research group-KYMERA, Konyang University Hospital, Daejeon, Republic of Korea; 3 Department of Rehabilitation Medicine, College of Medicine, Konyang University, Daejeon, Republic of Korea; 4 Department of Otorhinolaryngology-Head and Neck Surgery, College of Medicine, Konyang University, Daejeon, Republic of Korea

**Keywords:** Outcomes research, Drug safety

## Abstract

The increased risk of hearing loss with macrolides remains controversial. We aimed to systematically review and meta-analyze data on the clinical risk of hearing loss, tinnitus, and ototoxicity following macrolide use. A systematic search was conducted across PubMed, MEDLINE, Cochrane, and Embase databases from database inception to May 2023. Medical Subject Heading (MeSH) terms and text keywords were utilized, without any language restrictions. In addition to the electronic databases, two authors manually and independently searched for relevant studies in the US and European clinical trial registries and Google Scholar. Studies that involved (1) patients who had hearing loss, tinnitus, or ototoxicity after macrolide use, (2) intervention of use of macrolides such as azithromycin, clarithromycin, erythromycin, fidaxomicin, roxithromycin, spiramycin, and/or telithromycin, (3) comparisons with specified placebos or other antibiotics, (4) outcomes measured as odds ratio (OR), relative risk (RR), hazard ratio (HR), and mean difference for ototoxicity symptoms using randomized control trial (RCT)s and observational studies (case–control, cross-section, and cohort studies) were included. Data extraction was performed independently by two extractors, and a crosscheck was performed to identify any errors. ORs along with their corresponding 95% confidence intervals (CIs) were estimated using random-effects models. The Preferred Reporting Items for Systematic Reviews and Meta-analyses reporting guidelines for RCTs and Meta-Analysis of Observational Studies in Epidemiology guidelines for observational studies were followed. We assessed the hearing loss risk after macrolide use versus controls (placebos and other antibiotics). Based on data from 13 studies including 1,142,021 patients (n = 267,546 for macrolide and n = 875,089 for controls), the overall pooled OR was 1.25 (95% CI 1.07–1.47). In subgroup analysis by study design, the ORs were 1.37 (95% CI 1.08–1.73) for RCTs and 1.33 (95% CI 1.24–1.43) for case–control studies, indicating that RCT and case–control study designs showed a statistically significant higher risk of hearing loss. The group with underlying diseases such as multiple infectious etiologies (OR, 1.16 [95% CI 0.96–1.41]) had a statistically significant lower risk than the group without (OR, 1.53 [95% CI 1.38–1.70] *P* = .013). The findings from this systematic review and meta-analysis suggest that macrolide antibiotics increase the risk of hearing loss and that healthcare professionals should carefully consider this factor while prescribing macrolides.

## Introduction

Macrolides are antibiotics widely prescribed in various clinical specialties, including otolaryngology, internal medicine, and pediatrics, for the treatment of several infectious diseases^1–3^. However, concerns regarding the potential association between macrolides and hearing loss have persisted over time^4–9^. Although numerous randomized controlled trials (RCTs) have investigated this relationship, their results have been inconclusive^10–15^. Notably, a systematic review and meta-analysis conducted by Alsowaida et al.^16^ revealed that the statistical significance of the association between macrolides and hearing loss had not been established.

Nevertheless, clinicians in clinical medicine conduct studies to explore the potential of macrolides in causing hearing loss^17–20^. They frequently encounter patients who report tinnitus after being prescribed macrolides in clinical practice. In fact, Vanoverschelde et al. in 2021 reported a significant association between the use of macrolides and a 25% increase in the probability of tinnitus occurrence in a fully adjusted model^19^.

Consequently, concerns persist regarding the potential ototoxicity of macrolides. This concern is further amplified by increased societal interest in hearing loss, driven by increasing life expectancy worldwide^21–24^. Hearing loss not only poses challenges in daily life, but also has significant implications for individuals, including increased risks of depression and dementia^22,25,26^.

Since the last reported meta-analysis conducted on this topic in 2021, several studies have consistently reported an elevated risk of tinnitus or hearing loss associated with macrolides^18–20^. Therefore, further systematic reviews and meta-analyses are necessary. Accordingly, we performed this systematic review and meta-analysis encompassing all previously published studies on the association between macrolides and hearing loss to date. Additionally, through a subgroup analysis, we aimed to provide insights into aspects that may have been overlooked in previous meta-analyses, thereby offering a more comprehensive understanding of the topic.

## Materials and methods

This systematic review and meta-analysis is registered in the PROSPERO database (registration number: CRD42023426621) and conducted in accordance with the Preferred Reporting Items for Systematic Reviews and Meta-Analyses (PRISMA) statement^27^ and the Meta-Analysis of Observational Studies in Epidemiology (MOOSE) reporting guidelines^28^.

### Data sources and literature search

A comprehensive literature search was conducted in the PubMed, MEDLINE, Embase, and Cochrane databases using Medical Subject Headings (MeSH) terms and text keywords related to ototoxicity symptoms after macrolide antibiotic exposure, intervention (macrolide antibiotics), comparison (placebo or other antibiotics), and outcomes of ototoxicity symptoms from database inception to May 2023 (Supplementary Table S1). The search terms were categorized using Boolean operators (e.g., AND, OR, and NOT). The literature search was conducted regardless of the language or study design. Additionally, two independent researchers (SR Shim and JY Kim) manually and independently searched all relevant studies conducted in the US and European clinical trial registries and Google Scholar.

### Study selection

The study inclusion criteria were as follows: (1) studies including patients who had hearing loss [Common Terminology Criteria for Adverse Events (CTCAE) term, hearing impaired; MedDRA Code, 10019245], tinnitus (CTCAE term, tinnitus; MedDRA Code, 10,043,882), or ototoxicity symptoms after exposure to macrolide antibiotics, (2) intervention included prescription of macrolide antibiotics such as azithromycin, clarithromycin, erythromycin, fidaxomicin, roxithromycin, spiramycin, and/or telithromycin, (3) comparisons were specified as with a placebo or other antibiotics, and (4) outcomes were measured as odds ratio (OR), relative risk (RR), hazard ratio (HR), and mean difference for ototoxicity symptoms documented in RCTs and observational studies (case–control, cross-section, and cohort studies). In order to ensure data accuracy and relevance, certain studies, such as duplicate publications and publications that did not contain original data (review articles, case reports, conference abstracts, editorials, letters, and guidelines), were excluded from the analysis. Additionally, studies without comparison groups were also excluded from the analysis. Two investigators (SR Shim and JY Kim) independently analyzed the titles and abstracts as well as full-text articles according to the inclusion and exclusion criteria. A data extraction form was used independently by the authors to extract data. The final inclusion of articles was confirmed through an evaluation discussion involving all investigators. To ensure the integrity of the meta-analysis, references and data from each included study were meticulously verified to eliminate any overlapping data.

### Data extraction

Basic details about the studies (first author, year of publication, country, study design, number of patients, and duration of treatment), patient characteristics (age, sex, and disease), and technical aspects (treatments and controls) were extracted from the included articles using a predefined data extraction form. If a study included multiple treatment periods, the effect size was calculated. The final meta-analysis only included studies that provided comprehensive and complete information.

### Meta-analysis assessment of outcome findings and statistical analysis

The ORs, along with their 95% confidence intervals (Cls), were calculated for categorical variables^29,30^. The random-effects model created using the restricted maximum-likelihood (REML) estimator was employed to obtain the pooled overall ORs and 95% CIs for the outcomes^31^. The statistical heterogeneity was evaluated using the Cochran Q test and I^2^ statistic.

Each moderator was subjected to a meta-regression analysis for continuous variables (e.g., total number of patients, age, proportion of female sex, and duration of treatment) and a meta-analysis of variance for categorical variables (e.g., treatment based on disease type [multiple infection etiologies yes versus no], study design [RCT versus cohort versus case–control versus cross-sectional], country [Western versus Asian], control type [placebo versus no macrolide], method of hearing assessment [objective versus subjective], prescription of a single antibiotic [yes versus no], and prescription of azithromycin, clarithromycin, erythromycin, spiramycin, and/or telithromycin [yes versus no])^30^. An REML estimator was utilized to estimate the variance of true effects to analyze potential moderators.

A 2-sided *P*-value ≤ 0.05 or not contained of null value (OR = 1) within the 95% CI was considered significant. Analyses were conducted using R software version 4.2.1.

### Assessment of potential publication bias

A funnel plot was constructed to examine the potential presence of publication bias, utilizing the standard error as a measure of study size and ORs of macrolide antibiotic effects. In the absence of publication bias, the studies tend to exhibit a symmetrical distribution according to the combined effect size. In addition, we conducted Egger linear regression and method tests for assessing the publication bias, as well as Begg and Mazumdar rank correlation tests^30,32,33^.

### Quality assessment

The risk of bias (RoB) and methodological quality of the RCTs were assessed using the Cochrane Collaboration risk-of-bias 2.0 tool^34^. An RoB rating of high, low, or unclear was assigned to each domain during the assessment. The overall RoB was determined as follows: If all domains were rated as "low," the overall RoB was considered low. If at least one domain was rated as "some concerns," the overall RoB was considered to have some concerns. However, if at least one domain was rated as "high," or if more than two domains were rated as “some concerns,” the overall RoB was considered high.

The quality of case–control and cohort studies was assessed using the Newcastle–Ottawa Quality Scale (NOS)^35^. For each parameter, we used a star-based grading system. In the selection and outcome/exposure ascertainment categories, a study could receive a maximum of one star for each item. However, in the comparability category, a maximum of two stars could be awarded. The power of evidence regarding the assessment of benefits and drawbacks was presented based on specific conditions, indicating the quality of the evidence.

## Results

### Study selection

**Figure 1:**
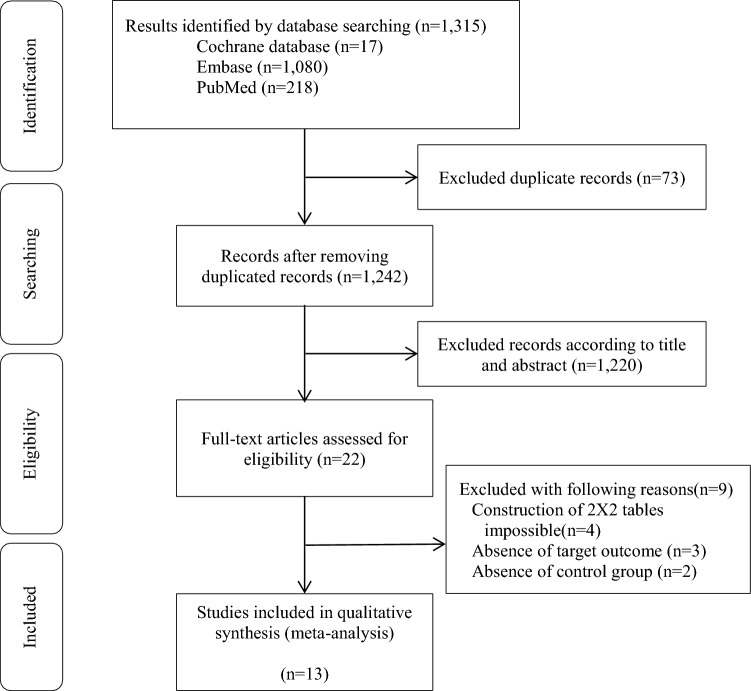
PRISMA study selection flow chart.

A total of 1,315 articles were identified during the initial search across different electronic databases, including PubMed (n = 218), Cochrane (n = 17), and Embase (n = 1080). Of these, 73 studies were excluded because of either containing overlapping data or appearing in multiple databases. After reviewing the titles and abstracts, 1220 studies were eliminated as they were found to be unrelated, trial registrations, or abstracts only. Among the remaining 22 full-text articles, 9 studies were further excluded for the following reasons: 4 because of the impossibility of constructing binary tables, 3 due to the absence of the target outcome, and 2 due to the absence of a control group. Ultimately, 13 studies met the selection criteria for qualitative and quantitative synthesis (Fig. 1).

We performed a systematic review and meta-analysis of these 13 studies that included a total of 1,142,021 participants. A detailed description of the differences and subject characteristics is provided in Table 1. Most studies were conducted in Western countries, while only two were conducted in Asia (Taylor^14^ and Tanaka^20^). The 13 included studies comprised 6 RCTs, 5 case–control studies, 1 cross-sectional study, and 2 cohort studies. The study by Hahn ^11^ had two overlapping types of study designs (RCT and case–control). The mean age of the participants ranged from 5.7 to 74 years, and the proportion of female sex ranged from 0 to 74.2%. The macrolide antibiotics used were azithromycin, clarithromycin, erythromycin, fidaxomicin, roxithromycin, spiramycin, and telithromycin. The controls were placebos or other antibiotics, and the average duration of treatment was 1 week to 24 months^7,10–15,17–20,36,37^.

**Table 1:** Characteristics of the included studies (n = 13).

Study	Country	Study design	Diseases	Average age (years)	Proportion of female sex (%)	Macrolide antibiotics	Controls	Duration of therapy	Method of hearing assessment
Swanson^7^	USA	Case–control	Community acquired pneumonia	62.9	0	E	Other intravenous antibiotics	1 week	Objective (PTA, SA, IA)
Saiman^15^	USA	RCT	Cystic fibrosis infected with Pseudomonas aeruginosa	20.2 ± 7.9	47.6	A	Placebo	6 months	Objective (NS)
Taylor^14^	Indonesia	RCT	Malaria prophylaxis	27	0	A	Placebo and doxycycline	5 months	Subjective
Grayston^13^	USA	RCT	Stable coronary artery disease	65	20.5	A	Placebo	24 months	Subjective
Albert^12^	USA	RCT	Chronic obstructive pulmonary disease	65 ± 9	40.8	A	Placebo	12 months	
Hahn^11^	USA	RCT and case–control	Adults with persistent asthma symptoms	45.6 ± 15.3	69.7	A	Placebo	3 months	
Altenburg^10^	Netherlands	RCT	Non-cystic fibrosis bronchiectasis	59.9 ± 12.3	58.9	A	Placebo	12 months	Subjective
Etminan^37^	Canada	Case–control	MIA	50.5 ± 10.6	56.4	A, C, E, T	No macrolide (matched by age, and calendared time to every patient without sensorineural hearing loss)		Objective (NS)
Alrwisan^36^	USA	Retrospective cohort	MIA	41	74.2	A	Amoxicillin	5–10 days	Objective (NS)
Tanaka^20^	Japan	Case–control	MIA		0	A, C, E, S, T	No macrolide		Subjective
Vanoverschelde^19^	Netherlands	Cross-sectional	MIA	68 ± 10	56	A, C, E, R, S	No macrolide		Objective (PTA)
Dabekaussen^18^	USA	Case–control	MIA	5.7 ± 4.9	38.2	A, C, E, F, T	Penicillin agents (matched by age, sex, and time)	12 months	Objective (NS)
Henkle^17^	USA	Retrospective cohort	bronchiectasis	74	67.9	A	Inhaled steroid	1 month	

USA, United States of America; RCT, randomized controlled trial; MIA, multiple infectious etiologies; A, azithromycin; C, clarithromycin; E, erythromycin; F, fidaxomicin; R, roxithromycin; S, spiramycin; T, telithromycin; PTA, Pure tone audiometry; SA, Speech audiometry; IA, Impedance audiometry; NS, Non-specific.

### Outcome findings from pairwise meta-analysis

**Figure 2:**
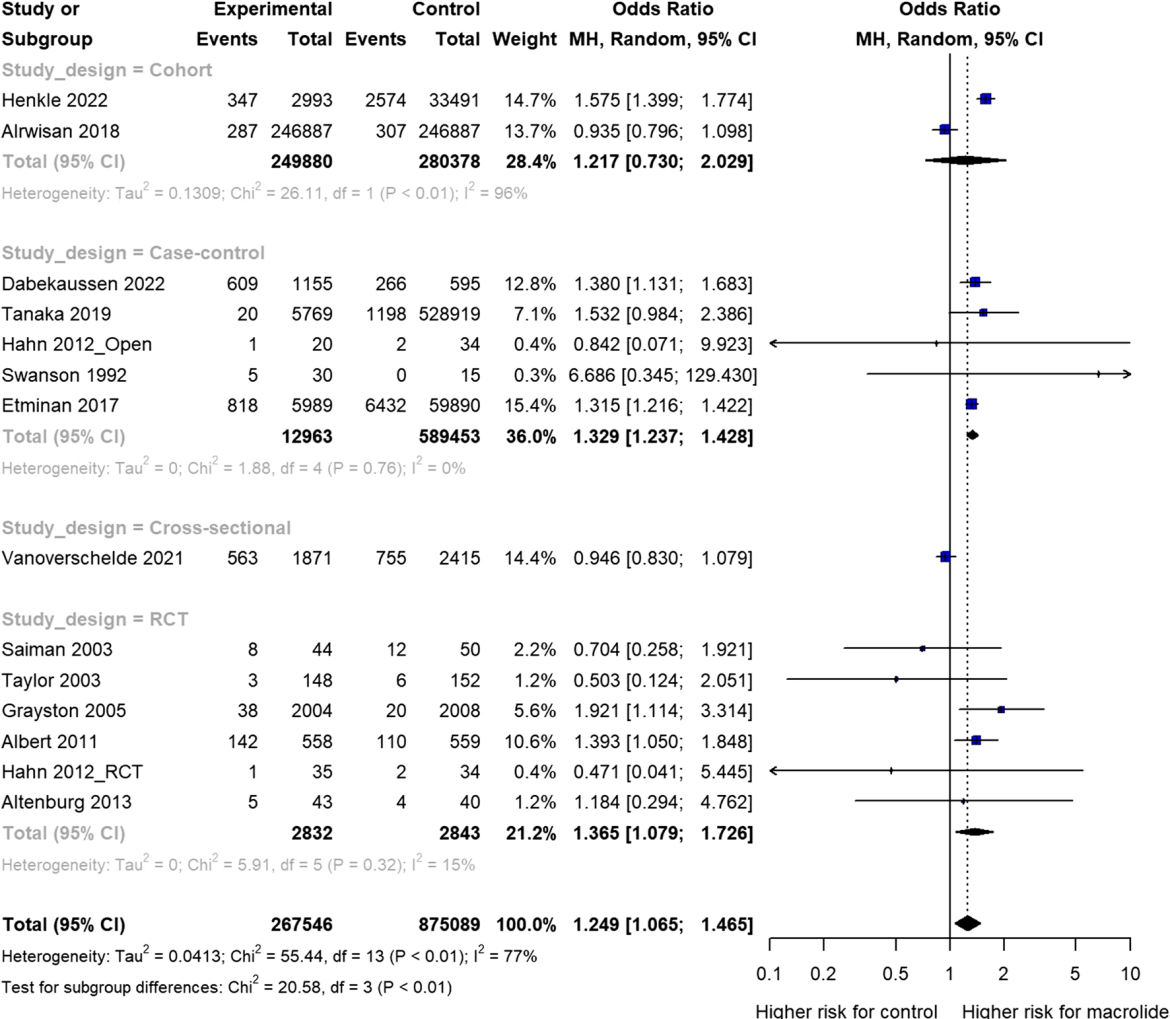
Odds ratio of hearing loss associated with macrolide antibiotic medication. The random-effects model created using the restricted maximum-likelihood estimator. CI, confidence interval. The black diamond shows the overall effect size.

The pooled OR for overall ototoxicity symptoms between macrolide and control groups was 1.25 (95% CI 1.07–1.47). The heterogeneity test resulted in a *P* value < 0.001 for Cochrane Q statistics, and Higgins’ I^2^ was 77%. Macrolide antibiotics were associated with a higher risk of hearing loss than placebos and other antibiotics. The subgroup analysis by study designs showed that the ORs were 1.37 (95% CI 1.08–1.73) in RCTs, 1.33 (95% CI 1.24–1.43) in case–control studies, 1.22 (95% CI 0.73–2.03) in cohort studies, and 0.95 (95% CI 0.83 to 1.08) in cross-sectional studies, indicating that the RCT and case–control study designs had a statistically significant higher risk (Fig. 2).

#### Moderator analyses

The study explored the potential moderating roles of specific variables through the application of meta-regression and meta-analysis of variance models. The outcomes of these analyses are detailed in Table 2. We found statistically significant differences among study designs (*P* < 0.001). The group with multiple infectious etiologies (OR 1.16, 95 %CI 0.96–1.41) had a significantly lower value than the group without (OR 1.53, 95% CI 1.38–1.70 *P* = 0.013). No significant differences were observed among the remaining covariates.

**Table 2:** Effects of moderators for ototoxicity related symptoms.

Variables	*k*	*β*	OR	95% CI		*P*
No. of total patients	14	0.000		0.000	0.009	0.539
Age	13	0.003		− 0.005	0.011	0.493
Proportion of female sex	13	− 0.507		− 1.539	0.525	0.336
Duration of therapy (months)	11	0.018		− 0.011	0.048	0.227
Disease type						0.013
Multiple infectious etiologies_Yes	5		1.162	0.961	1.406	
Multiple infectious etiologies_No	9		1.531	1.377	1.702	
Study design						< 0.001
RCT	6		1.365	1.079	1.726	
Cohort	2		1.217	0.730	2.029	
Case–control	5		1.329	1.237	1.428	
Cross-section	1		0.946	0.830	1.079	
Country						0.788
Western	12		1.245	1.050	1.475	
Asian	2		1.081	0.393	2.975	
Controls type						0.558
Placebo	7		1.359	1.075	1.717	
No macrolide	7		1.242	1.027	1.501	
Method of hearing assessment						0.122
Objective	7		1.153	0.968	1.374	
Subjective	4		1.543	1.115	2.134	
Mono antibiotic						0.499
Yes	9		1.152	0.846	1.570	
No	5		1.306	1.079	1.581	
Azithromycin						0.267
Yes	13		1.243	1.060	1.458	
No	1		6.686	0.345	129.430	
Clarithromycin						0.933
Yes	4		1.233	1.003	1.516	
No	10		1.251	0.960	1.630	
Erythromycin						0.419
Yes	6		1.315	1.086	1.592	
No	8		1.131	0.828	1.545	
Telithromycin						0.352
Yes	3		1.328	1.236	1.427	
No	11		1.181	0.932	1.497	
Spiramycin						0.634
Yes	2		1.148	0.722	1.823	
No	12		1.293	1.094	1.528	

*k*, number of effect sizes; *β*, regression coefficient; OR, odds ratio; *P*-value from meta-regression analysis using the restricted maximum likelihood; CI, confidence interval; RCT, randomized controlled trial.

### Publication bias

The statistical methods employed to detect publication bias or small-study effects are illustrated in Supplementary Figure S1. Individual ORs showed visually asymmetric graphics in funnel plots. The *P* values for the Begg and Mazumdar rank correlation test (*P* = 0.79) and Egger linear regression coefficient test (*P* = 0.62) indicated no evidence of publication bias or small-study effect in this meta-analysis.

#### Quality assessment

**Figure 3:**
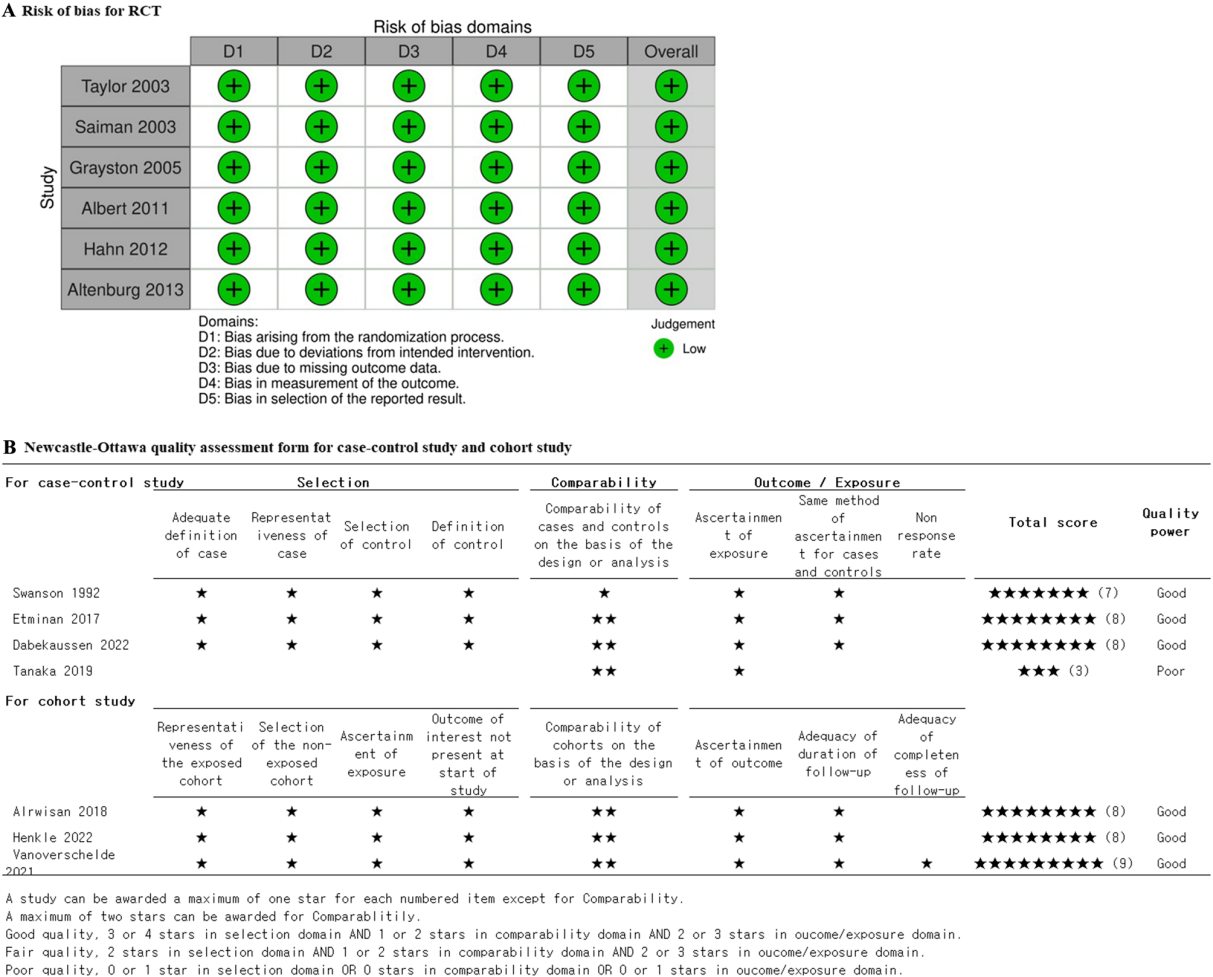
Risk of bias for randomized controlled trials and Newcastle–Ottawa quality assessment for observational studies.

We evaluated the 13 included studies using risk-of-bias 2.0 for RCTs and NOS for observational studies. In risk-of-bias 2.0, for D1, all studies were rated as “low”. In D2 to D5, all studies were rated as "low." The overall RoB was determined on the basis of these evaluations. Four studies were rated as "low" and two as "some concerns." All studies were ranked "good" (8–9 stars) except for the study by Dabekaussen^18^, which was rated as "poor" (3 stars) on the NOS (Fig. 3).

## Discussion

In this systematic review and meta-analysis, we found that the overall pooled effect size indicated a significantly increased risk of hearing loss associated with macrolide use, with an OR of 1.249 (95% CI 1.065–1.465). This finding contrasts with the results reported by Alsowaida et al. in 2021, where the estimated OR was 1.200 (95% CI 0.963 to 1.494), indicating no statistically significant association between macrolide use and hearing loss^16^. The discrepancy in results of meta-analyses conducted over the past few years can be attributed to the influence of several newly published clinical trials^17–19^. Recent clinical trials had large sample sizes and a high level of research design quality, suggesting that the findings of our meta-analysis hold a greater clinical significance.

Accumulating evidence from these recent clinical trials has contributed to a shift in the meta-analysis results, ultimately revealing a statistically significant association between macrolide use and an increased risk of hearing loss.

An intriguing aspect of our study was subgroup analysis, in which studies were categorized based on whether they focused on specific diseases or included a diverse range of conditions. Notably, subgroup analysis revealed significantly higher risk estimates, which adds an interesting dimension to the analysis. This finding raises the possibility that underlying conditions, such as multiple infectious etiologies, could influence the outcome of hearing loss.

Macrolides possess antimicrobial and anti-inflammatory properties, because of which they are widely prescribed^38,39^. Macrolides are predominantly employed for managing bacterial infections such as pharyngitis, otitis media, sinusitis, bronchitis, and community-acquired as well as atypical pneumonia. These antibiotics are especially useful for patients who have penicillin allergies^40,41^. In addition, macrolides are commonly utilized for eradicating *Helicobacter pylori* and managing sexually transmitted infections, specifically those that result from chlamydia and *Neisseria gonorrhea*^42–44^. They are additionally recommended for persistent inflammatory conditions, such as cystic fibrosis, asthma, and chronic obstructive pulmonary disease, due to their anti-inflammatory properties^41,45–47^.

Consequently, our meta-analysis included a diverse range of patients who were prescribed macrolides. Moreover, considering the distinct pathophysiologies of infections caused by different bacteria, the types of infectious diseases included in the analysis may have affected the risk of drug toxicity. Therefore, it is important to acknowledge the possibility that previous studies that did not specifically categorize patients based on their underlying conditions may have lacked appropriate adjustments for confounding variables. Consequently, the risk of hearing loss associated with macrolides may have been underestimated.

Considering these possibilities, more numbers of RCTs focusing on specific underlying conditions are required to accurately determine the causal relationship between macrolides and hearing loss. This would facilitate a more precise risk assessment based on specific types of infectious diseases.

The strengths of this study include the inclusion of all published studies on macrolide toxicity up to 2023. Moreover, to ensure a more focused analysis, we adopted analysis methods tailored to specific research subjects with the aim of excluding generic analytical approaches.

In our study, we specifically targeted published RCTs and conducted subgroup analyses using the REML, avoiding the use of generic analytical models. As a result, the OR for hearing loss associated with macrolides was estimated to be 1.363 (95% CI 1.080 to 1.720), demonstrating statistical significance. This finding contrasts with the results of a previous study in which a subgroup analysis was performed by pooling only six RCTs published between 2003 and 2013, and that showed no statistically significant association with an OR of 1.317 (95% CI 0.960–1.808).

These divergent results can be attributed to the following factors. The previous study employed the DerSimonian and Laird method, a widely used approach for estimating between-study variance in random-effects models. However, given the low event rates and small sample sizes in the RCTs included in our study, we opted for a more adjusted REML because it mitigates the downward bias that can occur when events are scarce. Therefore, the REML is recommended for such scenarios^48^.

Overall, the strengths of our study include its comprehensive inclusion of all published research on the toxicity of macrolides and the use of tailored analytical methods, particularly the application of REML, to analyze RCTs with low event rates. These methodological choices contributed to the result of a statistically significant association between macrolide use and hearing loss in our analysis.

This study has several limitations. First, a significant limitation was the inclusion of studies in which the control group received medications other than a placebo. For instance, in a retrospective cohort study conducted by Alrwisan et al., which was included in this meta-analysis, amoxicillin + clavulanate was used as a control for patients who were prescribed macrolides^36^. This introduced a potential confounding factor when comparing the effects of macrolides. Additionally, subgroup analyses were performed to compare studies including a placebo control group with those including other antibiotic controls. The OR was higher in studies with placebo controls (OR 1.359, 95% CI 1.075–1.717) than in studies with other antibiotics as controls (OR 1.242, 95% CI 1.027–1.501). While this suggests the possibility of some level of toxicity associated with antibiotics other than macrolides, it is important to note the limited number of studies in each group (seven in each group) and the non-significant *P* value of 0.558, indicating the need for caution when interpreting these findings. In addition, the diagnostic and assessment methods for hearing loss used in individual studies are an important factor in determining the overall reliability of a study^49^. Although the individual studies included in this study used multiple testing methods, there was no statistically significant difference between objective (OR 1.15, 95 % CI 0.96–1.37) and subjective (OR 1.54, 95% CI 1.12–2.13) methods (*P* = 0.122), suggesting that the assessment reliability of the collected studies is high (Table 2). Therefore, it is necessary to collect individual patient data in the future to approach the real facts through a comprehensive comparison with this meta-analysis.

Furthermore, because this meta-analysis relied on the aggregation of results from previously published papers, it was not possible to examine the individual characteristics included in each study. This limitation is regrettable as the risk of hearing loss associated with macrolide antibiotics may vary depending on the subcategories within the experimental group. Notably, Dabekaussen et al. in 2022 suggested that pediatric patients with sensorineural hearing loss (SNHL) had an increased likelihood of having received a macrolide prescription than a penicillin prescription^18^. Additionally, individuals diagnosed with SNHL more than 180 days after exposure were more likely to have received macrolides than penicillin-related medications.

Moreover, recent research has emphasized the potential influence of individual genetic factors on the responses to specific medications^50–54^. Therefore, future studies should investigate whether genetic predispositions make individuals more susceptible to the toxic effects of macrolides.

In conclusion, our study confirmed an increased risk of hearing loss associated with the use of macrolide antibiotics. Therefore, healthcare professionals should carefully consider these factors when prescribing macrolides. In particular, caution should be exercised while selecting macrolide antibiotics for patients with risk factors such as a family history of hearing impairment or preexisting hearing loss. Additionally, patients with hearing loss in only one ear should receive special attention when prescribing macrolides.

### Supplementary Information

Supplementary Information.

## Data Availability

This is a secondary data analysis using publicly available, existing data. Patients or the public were not directly involved in the design, or conduct, or reporting, or dissemination plans of our research. Data are contained within the article or supplementary material.
